# Method Development Progress in Genetic Engineering of Thraustochytrids

**DOI:** 10.3390/md19090515

**Published:** 2021-09-11

**Authors:** E-Ming Rau, Helga Ertesvåg

**Affiliations:** Department of Biotechnology and Food Science, NTNU Norwegian University of Science and Technology, N7491 Trondheim, Norway; e.m.rau@ntnu.no

**Keywords:** thraustochytrids, *Aurantiochytrium*, *Schizochytrium*, transformation, electroporation

## Abstract

Thraustochytrids are unicellular, heterotrophic marine eukaryotes. Some species are known to store surplus carbon as intracellular lipids, and these also contain the long-chain polyunsaturated fatty acid docosahexaenoic acid (DHA). Most vertebrates are unable to synthesize sufficient amounts of DHA, and this fatty acid is essential for, e.g., marine fish, domesticated animals, and humans. Thraustochytrids may also produce other commercially valuable fatty acids and isoprenoids. Due to the great potential of thraustochytrids as producers of DHA and other lipid-related molecules, a need for more knowledge on this group of organisms is needed. This necessitates the ability to do genetic manipulation of the different strains. Thus far, this has been obtained for a few strains, while it has failed for other strains. Here, we systematically review the genetic transformation methods used for different thraustochytrid strains, with the aim of aiding studies on strains not yet successfully transformed. The designs of transformation cassettes are also described and compared. Moreover, the potential problems when trying to establish transformation protocols in new thraustochytrid species/strains are discussed, along with suggestions utilized in other organisms to overcome similar challenges. The approaches discussed in this review could be a starting point when designing protocols for other non-model organisms.

## 1. Introduction

Thraustochytrids are heterotrophic marine microorganisms divided into ten genera [1]. They belong to stramenopiles, one of the most diverse eukaryotic phyla, known for groups such as diatoms, oomycetes and brown algae [1,2]. Thraustochytrids commonly inhabit oceans and sediments, especially in nutrient-rich areas, such as mangrove forests, where they grow on decomposing biological debris, and they play critical ecological roles for carbon recycling. Detailed biological features and classification have recently been extensively reviewed by others [3]. 

Thraustochytrids are well-known for storing up to 70% lipids (triacylglycerols) containing high amounts of ω3-polyunsaturated fatty acids (ω3-PUFAs), especially docosahexaenoic acid (DHA), produced by a dedicated polyketide synthase-like (PKS) enzyme complex [4,5,6]. DHA and other ω3-PUFAs, such as eicosapentaenoic acid (EPA), have substantial benefits for human health, including reducing the risks of cardiovascular, depressional, and neurodegenerative diseases [7,8,9]. As humans and most other vertebrates hardly synthesize DHA, it must be obtained by the diet. Currently, fish oil is the most used source for human and domesticated animals, including fish. Due to the limited fish stock available, alternative sustainable sources of DHA are needed, and thraustochytrids have been developed as commercial DHA-rich oils producers [10,11,12]. However, DHA from thraustochytrids is currently considered to be less competitive in low-cost markets [13]. On the other hand, some thraustochytrids produce other substances, such as squalene [10,14], carotenoids [15], extracellular enzymes [16], and extracellular polysaccharides [17], that potentially could be valuable biproducts.

Genetic engineering tools have become increasingly important in understanding specific metabolic pathways that could eventually be the prerequisite to create strains producing valuable materials in higher rates and titers [11,18,19]. Due to the advance and reduced cost of sequencing techniques, it is relatively easy to acquire genome sequences and identify gene targets for engineering. However, genetic method development of marine protists across taxa showed that no protocol could be universally applied [20]. Additionally, as will become apparent later, the established protocols for thraustochytrids all seem to be restrained to one or only a few strains. This is a general challenge for anyone working on non-model organisms; the transformation protocol needs to be designed for that particular strain. 

When a new strain is to be transformed, the challenge is how to find the first true transformant when one does not know which antibiotic resistance markers can be used, which promoters and terminators will work, how to transfer the DNA, or the efficiency of recombination in that particular strain. The multiple possible combinations of parameters make the method development on new strains complicated without proper feasibility clues based on previous experiences in the same genus. Still, experiences from related species may help. This review aims to compile and discuss the current transformation protocols and choice of DNA elements for thraustochytrids. Knowledge of what has been achieved for other microorganisms is included. Additionally, while this review focuses on thraustochytrids, it may also be read as an example of the different approaches that can be used to achieve gene knock-outs and gene knock-ins in other microorganisms.

## 2. Transforming DNA into the Cells: Methods and Considerations

**Figure 1:**
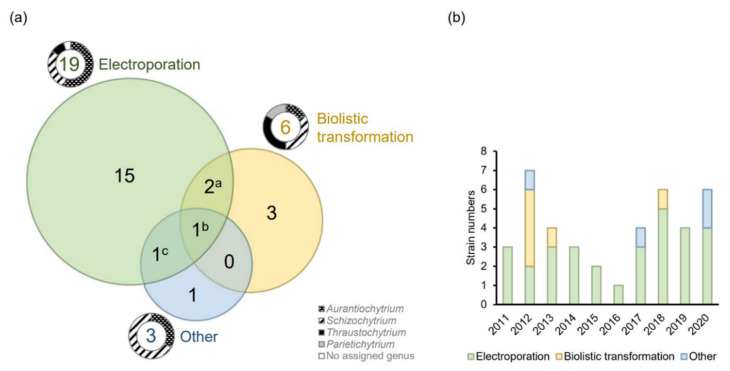
(**a**) The number of successfully engineered thraustochytrid strains by one or more methods (electroporation [20,21,22,23,24,25,26,27,28,29,30,31,32,33,34,35,36,37,38,39,40,41,42,43,44,45,46,47,48,49,50,51,52], biolistic transformation [5,27,53,54,55], and others [56,57,58,59]) (Tables S1 and S2). The doughnut pie charts indicate the proportion of different genera. Four strains have been transformed by more than one method: ^a^ *A. limacinum* mh018 and *T. aureum* ATCC 34304; ^b^ *Schizochytrium* sp. S31; ^c^ *Schizochytrium* sp. TIO1101; (**b**) The number of successfully transformed thraustochytrid strains and the methods applied in recent years. Any strain–method combinations are counted only once, even if they are used in several publications.

Electroporation, biolistic transformation, and *Agrobacterium*-mediated transformation (AMT) are used for many microbial cells, and all three methods have been successfully applied in thraustochytrids (Figure 1). 

During electroporation, electric pulses are applied to the cells, and pores may be generated in both the cell membrane and the nuclear membrane. Depending on the conditions, the pores can be reversible and non-lethal for the cell. When such pore-generation is used to facilitate the uptake of exogenous DNA, the process is called electrotransformation [60]. Biolistic transformation, or particle bombardment mediated transformation, uses high-pressure helium to inject DNA-coated non-reactive metal (tungsten or gold) particles into host cells. This has been applied to a wide variety of species [61]. The procedure is relatively simple, and it is the most widely used technique for genetic engineering in diatoms [62]. Still, the method has a relatively high cost. Its frequency of multiple copy random insertions is higher, and it causes more cell damage [61,63]. *Agrobacterium tumefaciens* is a plant pathogen that is widely used in generating transgenic plants, fungi, and microalgae. When *A. tumefaciens* infects a cell, part of its Ti plasmid, or binary vector, is integrated into the genome of the cell. AMT is relatively inexpensive. The DNA being transferred can be large (up to 150 kb) and with a single copy genome integration [64,65], which makes it possible to introduce an entire metabolic pathway.

It is important to note that there is a marked difference as to which thraustochytrid genera can be transformed by which method. Sakaguchi et al. [27] showed that electroporation is far more efficient than biolistic transformation in *Aurantiochytrium limacinum* mh0186, while biolistic transformation is more efficient than electroporation in *Thraustochytrium aureum* ATCC 34304. The electroporation protocol did not succeed at all in *Parietichytrium* sp. TA04Bb and *Schizochytrium* sp. SEK 579, both of which could be transformed by a particle gun. These results indicate that the fundamental biological features determining the success of any transformation method can be quite different between genera. As ectoplasmic nets, the unique ‘rhizoid-like’ cell membrane structures that extend from sub-cellular organelles are more apparent in *Parietichytrium* and *Schizochytrium* than *Aurantiochytrium* [3], the cellular structural features may affect the effectiveness of different transformation methods. This further suggests that one should test another of these principally different methods if the first one tested does not yield any results in a new microorganism.

### 2.1. Transformation by Electroporation

**Figure 2:**
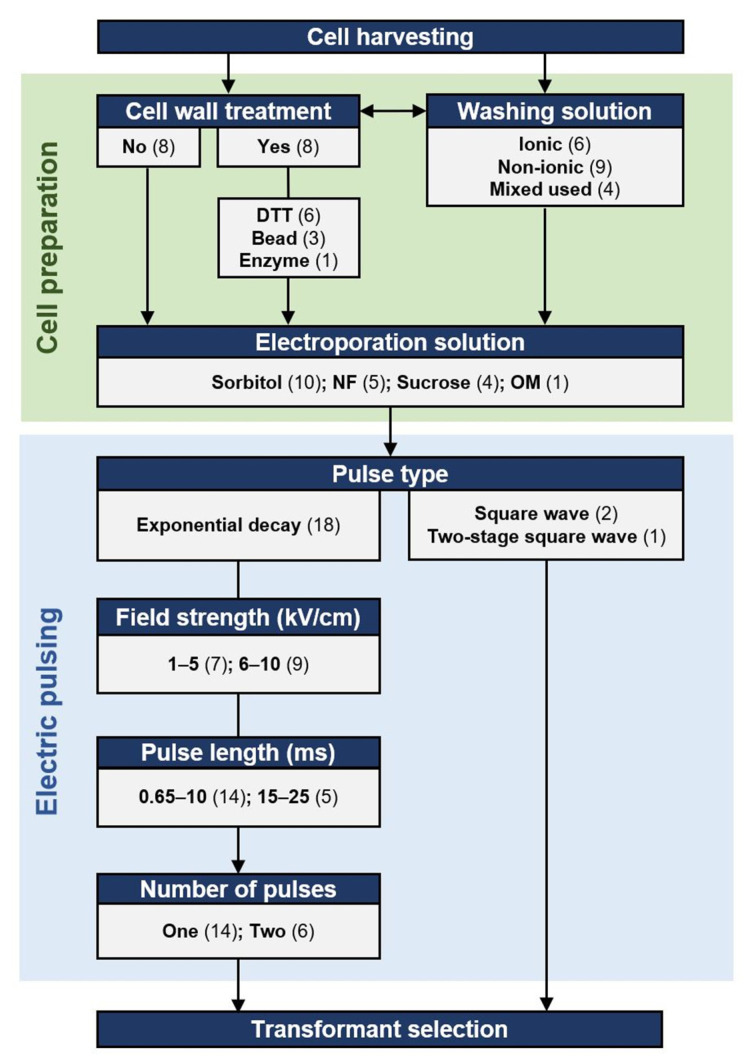
The parameters prevalence and the procedure scheme of transformation by electroporation in thraustochytrid strains. Numbers in parentheses, the number of strains that have used the parameter in at least one publication (Table S1); NF, Nucleofector^TM^ solution L; OM, OPTI-MEM^TM^ I; Ionic buffers include BSS, artificial sea water and phosphate buffer; Non-ionic, buffers include 50 mM Sucrose, 1 M Sorbitol, water, PEG 8000; Enzyme, 20 g/L pectinase and 20 g/L snailase in 7 M KCl.

In thraustochytrids, electroporation is the most common method for delivering DNA into cells (Figure 1). In the methods developed for thraustochytrid strains, both the treatment of cells prior to electroporation and the electroporation conditions themselves have been varied, as described in Figure 2. Exponential decay pulses and square wave pulses are the two most widely used pulse types for electrotransformation [66]. An exponential decay pulse is generated by exponentially reducing the initial voltage. Exponential decay pulse is described by two components: (1) electric field strength (kV/cm) is the electric potential difference (voltage) per unit distance between two electrodes; and (2) pulse length or time constant (ms), is equal to resistance multiplied by capacity [67]. The ionic strength and the volume of electroporation solutions affect the resistance of the samples so that the time constants are also affected. High ionic solutions with large volumes tend to have lower resistance, resulting in greater current and a higher probability of arcing and cell lethality. These parameters are further described below.

#### 2.1.1. Pulse Types, Numbers, and the Parameters

The known optimal values of pulse length and field strength vary among different species. For most microbes, pulse lengths lie within a range from 1 to 30 ms, and field strength from 1 to 20 kV/cm [60]. Exponential decay is the most used pulse type in thraustochytrids, with the applied field strengths vary from 1.8 to 10 kV/cm, while the set pulse lengths range from 0.65 to 25 ms (Figure 2). A square wave pulse is generated by quickly turning on and off a voltage, which is maintained at a stable level (constant field strength) in a short period of time (pulse length) [68]. Square wave pulses have been used to transform two thraustochytrid species (Figure 2, Table S1). 

A series of pulse parameters can be tested to find the optimal values. For *Aurantiochytrium* sp. SD116, the number of transformants decreased as the pulse length in the tested range increased [40]. Previous studies also show that a series of voltages should be tested to find the optimal field strength values during the new protocol [40,51]. For bacteria, optimal transformation results were often obtained by applying high field strengths with shorter time constants, or low field strengths with high time constants [67,69]. However, we found no similar correlations in the published protocols for thraustochytrids.

Most thraustochytrid protocols applied one exponential decay pulse per electroporation, while some applied two exponential pulses (Figure 2). In the microalgae *Chlamydomonas reinhardtii*, two pulses were required to introduce DNA into the cell, but only one pulse was needed to deliver DNA into the cell-wall-deficient mutant [70]. However, at least four thraustochytrid strains were engineered successfully with only one pulse without cell wall disruption (see Section 2.1.2), indicating that introducing DNA into thraustochytrid cells with intact cell walls does not require two exponential decay pulses. In addition, the transformation efficiency in *Aurantiochytrium* sp. SD116 increased about five times when the square wave pulse number increased from 30 to 50, followed by a five-times reduction when the number increased from 50 to 60 [40], suggesting that optimal pulse numbers can be narrow-ranged when applying electroporation with square wave pulses.

The initial voltage of an exponential decay pulse is high, to enhance cell permeabilization by pore generation, while the subsequential decayed low voltage part of the pulse contributes to electrophoretic transferring molecules into the cells [66]. As the voltages of the initial and later stage of an exponential decay pulse are related to one another, and cannot be adjusted independently, methods were developed with combinations of a first rapid high-voltage pulse followed by a longer low-voltage pulse in mammalian cells [71,72]. The low-voltage pulse was shown to contribute to the transfection efficiency when the plasmid concentration is low [73]. For thraustochytrids, *A. limacinum* SR21 has been transformed by two short, high voltage square pulses for poring cells, followed by one longer, lower voltage square pulse for molecule transferring using the NEPA21 electroporator [20]. This instrument can measure the resistance, allowing it to be adjusted to a specific range by altering cell volumes before pulsing (see Section 2.1.3), an informative function for further parameter optimization. 

High-throughput approaches can be performed to optimize pulse parameters. Through transforming the cells with fluorescently labeled DNA or cell-impermeable fluorescent molecules, the efficiency of delivering molecules to the cells can be measured by the number of fluorescent cells detected under different pulse conditions. This approach separates the process of transforming DNA from recombination and transcription of the inserted DNA. For example, the transformation of YOYO-1 labeled plasmid DNA was used to determine the permeability of yeast cells [74]. Similar approaches have been applied in microalgae, *Caecitelus* sp., *Nannochloropsis oceanica*, and *C. reinhardtii* through FITC-Dextran transformation [20,75]. However, fluorescently labeled DNA or cell-impermeable fluorescent chemicals can also accumulate in permeable but non-viable cells. In order to distinguish the permeable viable cells from permeable non-viable cells, Muñoz et al. [76] utilized two cell-impermeable dyes with different florescent emission wavelengths, Sytox Green and propidium iodide; these were mixed with the cells before the pulses and after the recovery from pulses, respectively, to measure transient cell permeability and viability independently. This method was successfully applied to find optimized pulse conditions in four microalgae species. A similar approach on thraustochytrids has not been reported to date. 

A more specific challenge for thraustochytrids is that their cells can co-exist in various growth stages, including medium vegetative cells, large multinucleated or sporangium cells, and small zoospores [1]. There are not only ambiguities between a single multinucleated cell, a single sporangium, and a cluster of multiple zoospores; the cleavages formed on the sporangium cells also decrease the roundness of cells to different extents. These various cell sizes and shapes make it difficult to distinguish individual cells by fluorescence microscopy or flow cytometry. They probably also are electrotransformed at different frequencies.

#### 2.1.2. Cell Wall Disruption or Removal

Cell walls are complex structures that generally contain various polysaccharides, lipids, and proteins. Disruption of cell walls has been shown to facilitate the uptake of molecules of the cells. For instance, the cell wall-less mutants of the microalgae *C. reinhardtii* could take up larger-sized molecules such as polysaccharides and proteins more efficiently [75,77]. In general, the electrotransformation efficiency of marine protists is relatively low without cell wall removal [20]. One example was the transformation efficiency in *C. reinhardtii*, shown to be up to ten times higher for the cell wall-less mutant than for the wild type [78].

Chemical treatments have been used to disrupt or remove cell walls. Dithiothreitol (DTT) contains two sulfhydryl groups, which can reduce the disulfide bridges of cell wall proteins to destabilize cell wall structures or even generate protoplasts. In yeast, cell wall porosity increased when the number of disulfide bridges of cell wall proteins decreased [79]. DTT-treatment resulted in the release of various proteins, glycoproteins, and polysaccharides from the outer cell wall layers of the yeast *Candida albicans* [80]. DTT has been applied to the transformation of multiple species, such as *Saccharomyces cerevisiae* and other fungi [81]. In thraustochytrids, DTT treatment is the most used method to disrupt or remove cell walls before electric pulses (Figure 2). Cell wall degrading enzymes, including pectinase and snailase (a mixture of many enzymes including cellulase, beta-glucuronidase, polygalacturonase, hemicellulase, protease, and pectinase), can also be used to prepare microalgae protoplast [82,83]. In *Schizochytrium* sp. PKU#Mn4, the two enzymes were applied to weaken the cell wall further after DTT treatment [46]. As thraustochytrid cell walls are generally composed of galactose-rich polysaccharides without cellulose [3], one would expect pectinase and polygalacturonase, but not the cellulase and hemicellulase, to play the major role in decomposing the cell walls in the protocol. However, using only these enzymes for disrupting cell walls remains to be tested in preparing thraustochytrid cells for electroporation. 

Cell walls can also be physically weakened by vigorously agitating the cells in the presence of glass beads, as demonstrated for yeast, the microalgae *Chlamydomonas* and thraustochytrids [32,84,85]. In thraustochytrid strain 12B and *A. limacinum* SR21, the transformation efficiency was improved by agitating with glass beads from nearly no transformants to 1.5–15 transformants/μg and 3–150 transformants/μg, respectively [32]. Hence, when establishing a thraustochytrid electroporation protocol, cell wall disruption is a parameter to consider.

#### 2.1.3. Effect of the Solutions Used to Prepare the Electrocompetent Cells

Typical electroporation solutions used on microbial cells are non-ionic osmotic stabilizers such as sorbitol and sucrose, to increase the cell survival rate [86]. In yeast, the transformation was more efficient with sorbitol as an electroporation solution than with sucrose [87]. Similarly, sorbitol is the most used electroporation solution for thraustochytrids, followed by sucrose (Figure 2), indicating that sorbitol can be a prioritized option in establishing the protocols. Although the absence of ions during electric pulse increases the cell viability, washing cells without ions could decrease the cell viability [88], which further complicates the selection of solutions. Even if the solutions used are non-ionic, the cells’ environment will not be entirely non-ionic due to incomplete washing. In thraustochytrids, both ionic (e.g., artificial seawater, BSS [51], and phosphate buffer) and non-ionic solution (H_2_O, sorbitol, and sucrose) has been used to wash cells (Figure 2). 

### 2.2. Transforming DNA into Thraustochytrid Cells by Non-Electroporation Methods

In thraustochytrids, biolistic transformation is the second most used approach (Figure 1a), but apparently is less used for thraustochytrids now (Figure 1b). However, it might just be that more groups have the equipment for studying the electrotransformable strains, and hence they are studied more. As mentioned earlier, some strains are only transformed by the biolistic method. Moreover, AMT has been used to engineer two *Schizochytrium* species (Table S2). Recently, a commercial kit originally developed for yeast transformation was successfully applied on *Aurantiochytrium* sp. YLH70 [56]. The protocol is significantly simpler than the methods mentioned previously. Although the detailed principle of the kit is not described, it seems to be related to the lithium cation-based chemical transformation that is commonly applied in yeast [81]. This implies that establishing protocols based on other existing protocols with more straightforward procedures might still be possible.

### 2.3. Other Strategies for Transferring DNA into Cells

Tremendous efforts have been dedicated from different research groups to broaden our skillsets in the genetic manipulation of thraustochytrids. However, several concerns could hinder generating systematic strategies based on the existing protocols. For instance, it would be beneficial if details on unsuccessful protocols and optimization strategies had been revealed, as demonstrated amiably by few studies [27,32,40,51], to reduce the unnecessary trials under limited times and resources. Moreover, it is unfortunately common to have transformation protocols reported without necessary details. This could affect the reproducibility and make it more challenging to interpret the optimal factors by protocol comparisons. Nevertheless, there are still genetic engineering strategies that could be tested on thraustochytrids, especially those that have been used on other stramenopiles and microalgae, or technologies developed recently.

*Escherichia coli* can transfer plasmids or episomes based on conjugative plasmids through conjugative bridges between the donor *E. coli* and the recipient cells, a process similar to AMT. Two plasmids are often used, including a cargo plasmid that contains the expression cassette and a helper plasmid (without the origin of transfer, *oriT*) that includes all genes required for transferring an *oriT*-containing plasmid. Conjugation does not require expensive equipment such as electroporators, and efficient conjugation-based genetic methods have been established for some diatom and green algae species [20,62,89]. For instance, the transformation efficiency of conjugation is higher than biolistic and electroporation in the diatom *Phaeodactylum tricornutum* [90,91], and a vector with 49 kb cargo DNA were introduced and maintained in *P. tricornutum* after conjugation [91].

Electroporation can also be developed in combination with digital microfluidics systems. Traditional ‘bulk’ electroporation usually applies voltage from hundreds to thousands of volts, which can cause water electrolysis in the part of solutions near the electrodes, resulting in local pH changes that reduce cell viability. In digital microfluidics systems, cells and DNA cassettes are encapsulated in tiny oily droplets before electric pulses with electrodes placed near the surfaces of the droplets so that the applied voltages can be largely reduced to 1 V–2 V though still being able to give an electric field strength in a range similar to bulk electroporation [92,93]. In addition, due to the relatively sizeable area-to-volume ratio of the droplets, the heat generated by the pulses can be more rapidly dissipated [94]. The droplet electroporation on microfluidic chips was found to have up to a thousand times higher transformation efficiency for the microalgae *C. reinhardtii* than the bulk electroporation using cuvettes [78,95], and can therefore be a promising system to test on transforming thraustochytrids and other microbes.

Cell-penetrating peptides (CPPs) are small peptides usually with less than 30 amino acids that show a remarkable ability to cross cell membranes and can transport biological materials intracellularly through non-covalent binding [96]. The TAT peptide (GRKKRRQRRRPQ) was the first CPP to be discovered. It is naturally part of the transactivator of transcription (TAT) protein of human immunodeficiency viruses. The TAT peptide has been used to facilitate the translocation of the dsDNA T-DNA into the microalgae *Chlorella vulgaris*, resulting in genomic integration of a DNA cassette. The peptide pVEC (LLIILRRRIRKQAHAHSK) derived from the murine vascular endothelial cadherin protein, has been used to transport Cas9-gRNA RNPs (see Section 3.3 and 3.4) into the microalgae *C. reinhardtii* for gene disruption. One advantage of both methods is the simplicity of the procedure, which only requires cells being treated with detergent or protease before or after mixing with the corresponding CPP-cargo [97,98]. Further investigation is required to determine the potential application of CPPs on the transformation of other species.

## 3. The Properties of the DNA Affect the Outcome of the Transformation

After the DNA has been transformed into the cell, it can be integrated in the genome at a specific location by homologous recombination (HR), utilizing the sequence homology-based DNA repair mechanism of the cells. Alternatively, DNA can be integrated in the genome at the location of random double-strand breaks (DSBs) by the non-homologous end joining repair (NHEJ) pathway. In most microalgae, random integration is significantly more efficient than HR, and widely applied to the genome engineering [62,99,100]. However, the site of the genomic integrations can affect the expression levels, and with random integration, one cannot utilize previous knowledge on the integration site. Moreover, non-target genes can be disrupted by random integration, resulting in misinterpretation of phenotypes unless the integration site is mapped [101]. Therefore, approaches such as introducing dedicated nucleases or interfering with NHEJ-specific enzymes have been proposed to enhance HR efficiency [102]. Exogenous genes without homology arms have been randomly integrated and expressed in at least nine thraustochytrid strains (Table S3). For four of these strains, random integration was shown to be more efficient than HR-based genome integration [27]. Episomes and plasmids, which can autonomously replicate extrachromosomally and carry expression cassettes, would also reduce the potential side effects from random genomic integration. Additionally, it is possible to eliminate extrachromosomal DNAs from the cell simply by removing the selection pressure from the culture.

### 3.1. The Presence and the Design of Homology Arms Affect Genome Integration

HR has been used for genome editing in prokaryotes and eukaryotes for decades, and it has, e.g., been exploited to express exogenous genes or disrupt existing genes in at least 20 thraustochytrid strains (Table S3). The most common design is to flank the cassettes with two homology arms, that is DNA with sequence homologous to a specific genome location, which can then integrate the cassette into genomes by HR. The efficiency of HR in cells could be enhanced when the length of the homology arms was increased [103,104]. A plasmid design for more straightforward construction work is to place only one homology arm next to the expression cassettes. The plasmid is then linearized by restriction enzymes cutting a site in the middle of the homology arm before transformation, and thus designed to integrate into the genome through a single crossover. This approach has been used in *T. aureum* ATCC 34304 and *A. limacinum* SR21 [20,27]. However, the HR efficiency of the one-homology-arm design is two times lower than the two-homology-arm design in *T. aureum* ATCC 34304 [27].

Endogenous promoter and terminator regions can be used directly as homology arms flanking the exogenous gene [39,55], the shorter cassettes could potentially increase DNA delivery efficiency. However, this would result in the replacement of the endogenous gene. 

### 3.2. The Structure and Quantity of DNA Affect Transformation Frequencies

The efficiency of HR also depends on whether DNA is linear or circular. Both linear and circular DNA molecules have been used to transform thraustochytrids (Tables S1 and S2). In *Schizochytrium* sp. CB15-5, there were no significant differences in the electrotransformation efficiency between introducing circular and linear DNA [51]. However, for the biolistic transformation of *T. aureum* ATCC 34304, only the linear, and not the circular, DNA molecule successfully generated transformants [27]. As DNA molecules are attached to beads, and then penetrate the cell by a pressurized gun in biolistic transformation, DNA structures are not expected to affect the ability of DNA to enter the cells. Moreover, Zhang et al., 2018 [46] performed electrotransformations in *Schizochytrium* sp. PKU#Mn4 with linearized DNA for genome integration by HR, but used a circular DNA vector for random genome insertion. This indicates that linear DNA could be more advantageous in performing HR in thraustochytrid cells. However, the paper does not quantitatively compare the two approaches.

In addition, adding more DNA could generate more transformants [60,87], which can be expected due to the increased possibility of DNA to contact with chromosomal DNA, resulting in higher genome integration rate. The amount of DNA added per electroporation ranges widely from 1 to 20 μg in thraustochytrids protocols. In *A. limacinum* SR21, adding one μg of DNA resulted in the highest electroporation efficiency, but the number of transformants increased from 44 to 68 when the DNA rose from 1 to 10 μg [20]. As DNA is not a costly material, and the goal of most studies is to obtain a high number of transformants, starting with a high amount of linear DNA seems the best strategy when a new strain is to be transformed. 

### 3.3. Strategies That Facilite Homologous Recombination

Inducing DSBs at specific sequences by sequence-specific nucleases can increase the efficiency of HR. Clustered, regularly interspaced short palindromic repeats (CRISPR)-associated protein (Cas) is a genome-editing technique widely used across species, with principles and features reviewed extensively by others [102]. With the assistance of site-specific guide RNA, DNA DSBs can be introduced by Cas9 or similar endonucleases. The DSBs can be repaired explicitly by homology-directed repair (HDR), resulting in predetermined deletions, insertions, or nucleotide changes determined by the added DNA where flanking arms are homologous to each side of the cut(s). Cas9 or Cas12 nuclease-induced DNA DSBs were shown to increase the efficiency of HR in the microalgae *C. reinhardtii* and diatom *Thalassiosira pseudonana* [105,106,107]. Recently, zeocin resistance cassettes were shown to be more efficiently integrated into the genome of *Aurantiochytrium* sp. RH-7A and *A. limacinum* SR21 with assistance from the CRISPR-Cas9 system using HDR [36]

Another approach to enhance HR might be to interfere with NHEJ-specific enzymes. For instance, does DNA ligase type IV ligate the nonhomologous DNA ends at the last step of NHEJ, and knockdown of this enzyme increased the rate of homologous recombination in *P. tricornutum* [103]. It is possible that HR efficiency in thraustochytrids can be increased by attenuating the expression of DNA ligase IV. However, thraustochytrid proteins are usually evolutionarily distant from those of model species, and a BLAST search in GenBank shows that most putative DNA ligase IV homologs encoded in the thraustochytrid genomes have less than 30% identity to those of *S. cerevisiae*, *Arabidopsis thaliana*, or *P. tricornutum* (unpublished). Hence, the function of putative proteins in the HR and NHEJ pathways in thraustochytrids needs to be experimentally verified, but the experimental data show them to be functional for genome engineering.

### 3.4. Application of Extrachromosomal DNAs

Extrachromosomal DNAs contain elements that function as centromere and origin of replication (or autonomously replicating sequences, ARS), and yeast-derived centromere and ARS and have been used to construct a replicating plasmid for *A. limacinum* OUC88, which was used to deliver the Cre-recombinase [30]. It is also possible to isolate elements that function as centromere or ARS from host genomes to support the autonomous replication of extrachromosomal DNAs [108]. 

In the recently established CRISPR-Cas9 method in thraustochytrids, the Cas9-gRNA Ribonucleoprotein (RNP) complex was directly electroporated into the cell to execute its function [36]. As Cas9-gRNA RNP is assembled in vitro by relatively costly gRNA and Cas9 proteins, an alternative strategy is to express both gRNA and the endonuclease after their genes are integrated into the genome, as already established in the diatoms *T. pseudonana* and *P. tricornutum* [109,110]. However, constitutive expression of Cas9 can cause re-editing (correction) of mutants, have a higher probability of off-target editing, and may be toxic to cells, which has been shown in microalgae such as *C. reinhardtii* [111]. To avoid this, Cas9 and gRNA can be expressed on an autonomously replicating episome so that the expression of Cas9 can be eliminated by removing the episome from the cell. This approach has been demonstrated in the diatom *P. tricornutum* [90], and is a promising approach to optimize the application of CRISPR-Cas9 genome editing in thraustochytrids. 

## 4. The Properties of the DNA Related to Gene Expression in Thraustochytrids

The gene expression level from an expression cassette will be affected by the choice of promoter and terminators, originated either endogenously, from other closely related species, or from other species. Constitutive promoters are commonly used to express heterologous genes with different strengths, while inducible promoters are advantageous at controlling the expression of toxic genes. Once transformation has been achieved, several strategies have been used to discover or design promoters that could be used to enhance transformation efficiency or control gene expression. Antibiotics selection is widely used to identify transformants expressing the corresponding antibiotic resistance gene. When the goal is gene-inactivation, one only needs to express a selectable marker gene if the organism is haploid, while diploid organisms would necessitate two marker genes for complete inactivation. Expanding the number of antibiotics applicable for transformant selection can boost the genetic engineering capability, as multiple genes of an organism can be disrupted by different antibiotic-resistance cassettes through HR. On the other hand, in most cases one would want to express one or more additional genes. Then, each gene would need their own promoter and terminator, resulting in long DNA cassettes for transformation. Approaches such as utilizing self-cleaving peptides (Section 4.3) or co-transforming separate single gene-containing DNA molecules can reduce DNA size when co-expressing antibiotic-resistance gene with gene of interests (GOIs), which potentially enhance DNA delivery.

### 4.1. Promoters and Terminators Used for Controlling Cassette Expression Level

**Figure 3:**
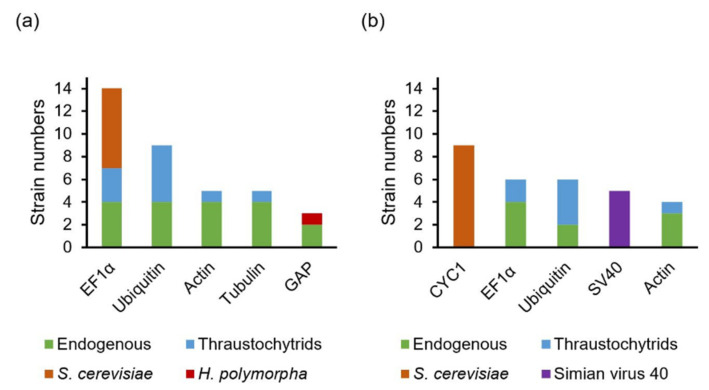
The prevalence of constitutive promoters (**a**) and terminators (**b**) used in thraustochytrids genetic engineering. Strain numbers: the number of strains that have used the promoter/terminator in at least one publication (Tables S3 and S4).

In yeast, the elongation factor 1-alpha (EF1α) promoter is one of the strongest constitutive promoters used [112,113], and the cytochrome c1 (CYC1) terminator is among the three most used terminators [114]. In thraustochytrids, the EF1α promoter and terminator are one of the most frequently used endogenous promoters and terminators, while *S. cerevisiae*’s EF1α (TEF1) promoter and CYC1 terminator are the most frequently used non-endogenous promoter and terminator (Figure 3 and Table S4). In *Schizochytrium* sp. CB15-5, transformants were more efficiently generated by expressing the zeocin resistance cassette with the endogenous EF1α promoter-terminator than with the endogenous actin promoter-terminator, followed by the endogenous glyceraldehyde 3-phosphate dehydrogenase (GAP) promoter-terminator. The number of transformants with the endogenous EF1α promoter-terminator was up to 15 times higher than that with the endogenous GAP promoter-terminator [51]. Recently, a promoter activity assay was established in *Schizochytrium* sp. S31 and the tested promoters, here listed by descending promoter strength, were glucose-repressible gene (ccg1) of *Neurospora*, TEF1, endogenous EF1α, and endogenous ubiquitin [48]. Hence, the EF1α and the ccg1 promoters are presumably more secure options for establishing protocols on new strains. There are also no indications that endogenous promoters are preferable in thraustochytrids. However, these comparisons of promoter strength were all performed in the *Schizochytrium* genus. Whether the trends are similar in *Aurantiochytrium* and other genera is unknown.

Strong promoters have been determined based on a transcriptomic analysis in the diatom *P. tricornutum* [115]. One approach has also been to transform promoter-less marker genes to subsequently identify the marker genes’ upstream sequence with strong expression in the transformants of the microalgae *C. reinhardtii* [116]. However, this approach only applies to those strains with established transformation protocols. There are also limited choices of marker genes that are able to generate easy-to-screen phenotypes when they are expressed. Finally, it is possible to generate synthetic promoters by error-prone PCR or rational design, which has been reviewed in detail [94,95]. Similarly, stronger terminators can also be identified by screening fluorescent reporter proteins with a combination of thousands of terminators [117]. 

Compared to constitutive promoters, significantly fewer thraustochytrids were engineered with inducible promoters, including the galactose-inducible galactokinase (GAL1) promoter of *S. cerevisiae* [30], the ethanol-inducible alcohol dehydrogenase I (AlcA) promoter of *Aspergillus nidulans* [57,58], and the methanol-inducible alcohol oxidase I (AOX1) promoter of *Pichia pastoris* [48] (Table S3). Although the strength of the induced AOX1 promoter was discovered to be weaker than TEF1, the AOX1 promoter had no detectable promoter activity in the absence of methanol [48], indicating its great potential for controlling the expression of a toxic gene in thraustochytrids.

### 4.2. Antibiotics Resistance Genes for Transformant Selection in Thraustochytrids

G418, zeocin, and hygromycin are the most frequently used antibiotics in thraustochytrids. However, the concentrations needed vary largely between different strains (Table 1). For example, the zeocin concentration applied for selecting transformants in *Thraustochytrium* sp. ONC-T18 is more than 100 times higher than that used for *Schizochytrium* sp. HX-308 [55]. The antibiotic concentration used in different protocols for the same strain also varies (Table 1). One possibility is that one tends to choose a concentration somewhat higher than the minimum inhibitory concentration (MIC). Additionally, as the transformation process may result in different cell fragility levels, cells can have different levels of antibiotic tolerance. Furthermore, the density of cells growing on the selective agar media can alter the resistance. For the yeast *P. pastoris*, it has been shown that dead cells can absorb zeocin and reduce the perceived concentration, allowing untransformed cells to grow as false-positive colonies [118]. A similar phenomenon also existed when we attempted to select *Aurantiochytrium* sp. T66 transformants by zeocin (unpublished), potentially aggravated by the cells’ clustering-prone phenotype. Overall, although G418, zeocin, and hygromycin can be prioritized for new protocol establishments, it is difficult to estimate the concentration for a new strain based on previous research on other strains. The MIC must always be determined using cell concentrations similar to those that will be utilized when plating out transformed cells. When establishing a protocol, it may be worthwhile to plate transformants on a couple of different antibiotics concentrations to avoid both false negatives and the risk of exceeding the resistance level for transformed cells.

Expanding the number of antibiotics applicable for transformant selection is especially critical for gene inactivation in diploid thraustochytrid strains such as *T. aureum* ATCC 34304, as each allele must be disrupted separately to inactivate the gene function completely [27,54]. Potentially applicable antibiotics for thraustochytrids can be selected from those applied on other stramenopiles, including phleomycin, nourseothricin, puromycin, and formaldehyde [20]. As both zeocin and phleomycin belong to the bleomycin family with the same corresponding resistance gene, phleomycin can be tested on those strains that fail to be selected by zeocin without changing the resistance cassette. However, as antibiotic resistance of thraustochytrids is among the highest compared to other marine protists, expanding the number of applicable antibiotics is expected to be challenging [20]. 

Finally, removal of the resistance gene from the genome by Cre/*lox*P system was demonstrated in *A. limacinum* OUC88 [30]. This method can reduce the number of different antibiotics needed to generate a series of genome integrations and, therefore, it would be worthwhile to explore its application on more thraustochytrid strains.

### 4.3. Expression of Multiple Genes

Cassettes containing a GOI and antibiotic resistance gene have been expressed in at least 18 thraustochytrid strains (Table S3). In most of these cassettes, each gene was flanked with an individual pair of promoter and terminator. These were different for each gene on the cassette, potentially intended to avoid intramolecular HR that can truncate the plasmids [119]. One exception is that two copies of the same phosphoglycerate kinase (PGK) promoter were used on the same cassette without negative consequences mentioned in *A. limacinum* OUC168 [29].

As multiple promoters and terminators on the same cassette significantly increase the cassette size, it potentially reduces DNA delivery efficiency and limits the number of genes expressed on a single cassette. An alternative approach is to connect multiple genes with 2A linker sequences as a single multicistronic transcription unit, driven by a single pair of promoter and terminator. 2A linker sequences are 18–22 amino acids viral origin peptides that introduce cleavage of polypeptides during translation in eukaryotic cells [120]. Picornavirus 2A (P2A) peptides have been used in *Aurantiochytrium* sp. SK4 [43], *Thraustochytrium* sp. ONC-T18 [55], and *A. limacinum* SR21 [52], while foot-and-mouth disease virus 2A (F2A) peptides have been used in *Aurantiochytrium* sp. SD116 [41]. In *Aurantiochytrium* sp. SK4, three genes were linked via P2A sequences [43], demonstrating the possibility for co-expressing several genes of interest. The efficiency of 2A cleavage may depend on the 2A peptide or the strain that is used. In *Aurantiochytrium* sp. SD116, the proteins that contain F2A peptides were nearly 100% cleaved [41]. However, in *Thraustochytrium* sp. ONC-T18, the proteins that comprise P2A peptides were only partially cleaved [55]. 

Another approach that could reduce the cassette sizes for transformation would be to put the antibiotics resistance gene and the genes of interest on separate DNA molecules and transform them simultaneously. Although it is apparent that not all antibiotic-resistant transformants can be expected to express the gene from the other DNA molecule, this approach has been applied in the diatom *P. tricornutum* combined with biolistic transformation and around 60–70% of the transformants expressed genes originated from both plasmid [121]. Studies using this approach on thraustochytrids have not yet been published. 

## 5. Conclusions

Most eukaryotic microorganisms have not been identified to date, and among those known to have biotechnologically interesting properties, only a small fraction have been transformed. This review compiles the current knowledge that is either used or have the potential to be used in one group of marine microorganisms that, evolutionarily, is quite different from other organisms as recently shown [122]. However, the approaches and elements used are similar to those used in other species, such as yeast, green microalgae, and diatoms, and hence will be relevant to consider for eukaryotic microorganisms in general.

Thraustochytrids are becoming increasingly interesting for sustainable biomanufacturing. Thus far, only some thraustochytrids have been successfully engineered by protocols tailored to the species and strains of interest. Still, developing genetic tools in new thraustochytrid strains presents significant challenges. Although methods such as biolistic transformation and AMT have been used, electroporation is the most widely used method for delivering DNA into thraustochytrids cells, with experimental setup of electric pulses, cell wall treatment, and the solutions as most common factors to be considered. Moreover, the properties of expression cassettes, including promoters, terminators, homology arms, antibiotics resistance genes, self-replicating sequences, linkers, and vector structure and quantity, play important roles in heterologous expression experiments. Further systematic studies to narrow down the critical parameters involved in thraustochytrid genetic engineering methods are needed to effectively overcome fundamental barriers of protocol establishment. Emerging tools such as CRISPR-Cas, conjugation, microfluidics, and CPPs may open up new solutions to simplify the engineering process establishment in thraustochytrids, as well as other microorganisms.

## Figures and Tables

**Table 1:** Antibiotics used for selecting transformants of thraustochytrids.

Strain	Zeocin	Hygromycin	G418	Blasticidin	Other	Reference
*A. limacinum* F26-b		2000	500			[21,22,23]
*A. limacinum* mh0186	500 *	1000; 2000 *	500	1200	500 (neomycin)	[24,25,26,27,28]
*A. limacinum* OUC168	5				100 (chloramphenicol)	[29]
*A. limacinum* OUC88	5				100 (chloramphenicol)	[30]
*A. limacinum* SR21	30; 50; 100	200	500			[20,31,32,33,34,35,36,52]
*Aurantiochytrium* sp. KRS101					30 (cycloheximide)	[37]
*Aurantiochytrium* sp. MP4	50					[38]
*Aurantiochytrium* sp. PKU#SW7		500	500			[39]
*Aurantiochytrium* sp. RH-7A	100					[36]
*Aurantiochytrium* sp. SD116	30; 50; 100	500 *	50 *; 50		100 *(anhydrotetracycline)	[40,41,42]
*Aurantiochytrium* sp. SK4	50					[38,43]
*Aurantiochytrium* sp. YLH70	15					[56]
*Parietichytrium* sp. TA04Bb		2000	500	800		[27]
*Schizochytrium* sp. S31	40; 50		100		50 (bleomycin); 250 (cefotaxime); 50 (paromomycin)	[5,47,48,53,57,58]
*Schizochytrium* sp. HX-308	1.5; 20					[44,45]
*Schizochytrium* sp. PKU#Mn4			800			[46]
*Schizochytrium* sp. TIO01	100					[49]
*Schizochytrium* sp. TIO1101			300			[50,59]
*Schizochytrium* sp. SEK 579		2000	500			[27]
*Schizochytrium* sp. CB15-5	20					[51]
Thraustochytrid strain 12B			500			[32]
*T. aureum* ATCC 34304		2000	1000; 2000	200–400		[27,54]
*Thraustochytrium* sp. ONC-T18	250	400				[55]

Each number represents the minimal concentration (µg/mL) used on agar for transformant selection in the reference; * MIC identified in the reference that was not used in transformant selection. The references of each used concentration are shown in Table S5.

## Data Availability

Not applicable.

## Supplementary Materials

The following are available online at https://www.mdpi.com/article/10.3390/md19090515/s1, Table S1: Electroporation conditions used in thraustochytrid transformation, Table S2: Non-electroporation transformation methods used in thraustochytrids, Table S3: Promoters and terminators used for thraustochytrids genetic engineering as well as the insertion type and the expressions of GOIs in thraustochytrids genetic engineering, Table S4: The prevalence of constitutive promoters and terminators used in thraustochytrids genetic engineering, and Table S5: Antibiotics used for selecting transformants of thraustochytrids with detailed information regarding to the reference of each concentration.
