# Supplementary material for: Method Development Progress in Genetic Engineering of Thraustochytrids
Source: Mar Drugs. 2021 Sep 11;19(9):515. doi: 10.3390/md19090515 (PMC8467673; doi:10.3390/md19090515)
Supplement: Supplementary file 1 [file marinedrugs-19-00515-s001.zip › marinedrugs-1301108-supplementary.pdf]

Table S1. Electroporation conditions used in thraustochytrid transformation.

Strain	Washing solution	Cell wall treatment	Electroporation solution	DNA structure	Pulse length, ms (mF × Ω)	Field strength (kv/cm)	Pulse numbers	Efficiency	Notes	Reference
Aurantiochytrium limacinum F26-b	ASW	No	OPTI-MEM™ I	L	5	8.5	2	n/a		[21]
	water	No	NF	L	2.5; 10	7.5	2	n/a		[22]
	water	No	NF	C	2.5	7.5	2	n/a		[23]
Aurantiochytrium limacinum mh0186	n/a	n/a	NF	L	2.5	7.5	2	n/a		[24]
	ASW	No	NF	L	5	7.5	2	n/a		[25]
	ASW	No	NF	L	2.5	6	2	n/a		[26]
	ASW	No	NF	L	2.5	7.5	2	160	Highest efficiency reported	[27]
	n/a	n/a	n/a	n/a	n/a	n/a	n/a	n/a		[28]
Aurantiochytrium limacinum OUC168	n/a	n/a	n/a	L	10	1.8	1	n/a		[29]
Aurantiochytrium limacinum OUC88	Water; Sorbitol	No	Sorbitol	C	10	1.8	1	n/a		[30]
Aurantiochytrium limacinum SR21	BSS, Sucrose	No	Sucrose	L	25	2.25	2	44	This efficiency was the best among the tested conditions, including protocols done by two-staged square wave pulses (NEPA21)	[20]
	BSS, Sucrose	No	Sucrose	L	n/r	n/r	3	n/a	Two-staged square wave pulses (NEPA21)	[52]

	ASW	No	NF	L	2.5	7.5	2	n/a		[31]
	50% ASW; Sucrose	Beads	Sucrose	L	5	5	1	30-150	The efficiency was nearly zero without treating beads	[32]
	Water; 0.1 M PB pH 6.5	DTT	Sorbitol	L	n/a	n/a	n/a	n/a	Square wave pulses	[33]
	n/a	n/a	n/a	n/a	n/a	n/a	n/a	n/a	Square wave pulses by custom-built electroporator	[34]
	Water	DTT	Sorbitol	L	6	10	1	n/a		[35]
	ASW	No	NF	n/r	2.5	7.5	1	n/a	Electroporation of Cas9-gRNA RNP complex	[36]
Aurantiochytrium sp. KRS101	Water	DTT	Sorbitol	L	25	10	1	7-55	The efficiency varied according to the antibiotics (Cycloheximide) selection concentration	[37]
Aurantiochytrium sp. MP4	n/a	n/a	n/a	L	15	n/a	1	n/a	50–100 colonies/plate by unknown amounts of DNA	[38]
Aurantiochytrium sp. PKU#SW7	n/a	n/a	Sorbitol	C	2.5	3	2	n/a		[39]
Aurantiochytrium sp. RH-7A	ASW	No	NF	n/r	2.5	7.5	1	n/a	Electroporation of Cas9-gRNA RNP complex	[36]
Aurantiochytrium sp. SD116	Sorbitol	DTT (buffer A)	Sorbitol (buffer B)	L	0.1	6	50	30-80	Square wave pulses by custom-built electroporator Multiple parameters were screened	[40]
	Sorbitol	DTT (buffer A)	Sorbitol (buffer B)	L	0.1	6	50	n/a	Square wave pulses by custom-built electroporator	[41, 42]
Aurantiochytrium sp. SK4	n/a	n/a	n/a	L	15	n/a	1	n/a	50–100 colonies/plate by unknown amounts of DNA	[38, 43]
Schizochytrium sp. HX-308	Water; Sorbitol	No	Sorbitol	L	10	3.75	1	n/a		[44]

Schizochytrium sp. PKU#Mn4	Water; Sorbitol	DTT; Enzymes	Sorbitol	L/C	25	10	1	n/a	The cassettes with 18S homology arms were linearized	[45]
Schizochytrium sp. S31	5.0% PEG 8000; 114% ASW; Sucrose	Beads	Sucrose	n/a	10	10	1	n/a		[46]
	Water; Sorbitol	No	Sorbitol	L	10	7.5	2	n/a		[47]
Schizochytrium sp. TIO01	Water; Sorbitol	DTT	Sorbitol	L	10	9	1	>100		[49]
Schizochytrium sp. TIO1101	20 mM PB	DTT	Sorbitol	L	4.5	n/a	1	n/a		[50]
Schizochytrium sp. CB15-5	BSS; Sucrose	n/a	Sucrose	L	0.65	2.5	1	0.5-7, 1.2-50, 0.5-3	Different efficiencies by using Actin, ef1α, GAP promoter, respectively	[51]
Thraustochytrid strain 12B	50% ASW; Sucrose	Beads	Sucrose	L	5	5	1	1.5-15	The efficiency was zero without treating beads	[32]
Thraustochytrium aureum ATCC 34304	ASW	No	NF	L	2.5	7.5	2	n/a	The efficiency was described as extremely low	[27]

The waveform was exponential decay if not specially mentioned; Efficiency, number of transformants per ug of cassette DNA; ASW, artificial sea water (1.75% (weight/volume) sea salt); n/a, not available; NF, Nucleofector™ solution L; Sorbitol, 1 M sorbitol; BSS, 10 mM KCl, 10 mM NaCl, and 3 mM $CaCl_2$; Sucrose, 50 mM sucrose; PB, phosphate buffer; n/r, not relevant; Buffer A, 0.6 M sorbitol, 0.1 M LiAc, 10 mM DTT, pH 7.0; Buffer B, 10 mM K2HPO4, 5 mM MgCl2, 1 M sorbitol, pH 7.4; Enzymes, 20 g/L pectinase and 20 g/L snailase in 7 M KCl; L, linear; C, circular.

Table S2. Non-electroporation transformation methods used in thraustochytrids.

Strain	Transformation method	DNA structure	Efficiency	Reference
Aurantiochytrium sp. YLH70	Frozen-EZ Yeast Transformation II	L	n/a	[56]
A. limacinum mh0186	Biolistic	L	Extremely rare	[27]
Parietichytrium sp. TA04Bb	Biolistic	L	50	[27]
Schizochytrium sp. S31	AMT	n/r	n/r	[57]
	AMT	n/r	n/r	[58]
	Biolistic	C	>2	[53]
	Biolistic	C	125-12.5	[5]
Schizochytrium sp. TIO1101	AMT	n/r	n/r	[59]
Schizochytrium sp. SEK 579	Biolistic	L	46	[27]
T. aureum ATCC 34304	Biolistic	L	190	[27]
	Biolistic	L	n/a	[54]
Thraustochytrium sp. ONC-T18	Biolistic	L	n/a	[55]

Efficiency, number of transformants per ug of cassette DNA; n/a, not available; AMT, *Agrobacterium tumefaciens*-mediated transformation; n/r, not relevant; L, linear; C, circular.

Table S3. Promoters and terminators used for thraustochytrids genetic engineering as well as the insertion type and the expressions of GOIs in thraustochytrids genetic engineering.

Strain	Promoter	Terminator	Insertion type
A. limacinum F26-b[a]	EF1α (n/a) [21, 22]; Ubiquitin (*T. aureum*) [21-23]	EF1α (n/a) [22]; Ubiquitin (n/a) [21]; SV40 [21-23]	HR [21-23]; Random [21, 22]
A. limacinum mh0186[a]	EF1α (*T. aureum*) [24-28]; Ubiquitin (*T. aureum*) [24-28]	EF1α (*T. aureum*) [24-28]; Ubiquitin (*T. aureum*) [24-28]	HR [25]; Random [24-28]
A. limacinum OUC168[a]	PGK (*Sc*) [29]	CYC1 [29]	HR [29]
A. limacinum OUC88[a]	EF1α (*Sc*) [30]; PGK (*Sc*) [30]; GAL1(*Sc*) [30]	CYC1 [30]	HR [30]
A. limacinum SR21[a]	EF1α (*Sc*) [33-35]; GAP [20, 52]; Ubiquitin (*T. aureum*) [31]; EF1α (*T. aureum*) [31]; EF1α (12B) [32]; Actin (RH-7A) [36]	CYC1 [33-35]; GAP [20, 52]; Ubiquitin (*T. aureu*m) [31]; EF1α (*T. aureum*) [31]; EF1α (12B) [32]; Actin (RH-7A) [36]	HR [20, 32-35, 52]; Random [31]
Aurantiochytrium sp. KRS101[a]	GAP (*Hp*) [37]	AOX (*Hp*) [37]	HR [37]
Aurantiochytrium sp. MP4[a]	Tubulin (SK4) [38]	SV40 [38]	HR [38]
Aurantiochytrium sp. PKU#SW7	PH [39]; DH [39]	PH [39]; DH [39]	HR [39]
Aurantiochytrium sp. RH-7A	Actin [36]	Actin [36]	n.r.
Aurantiochytrium sp. SD116[a]	EF1α [40]; Actin [40]; EF1α (*Sc*) [41, 42]; Tubulin [42]	EF1α [40]; Actin [40, 42]; CYC1 [40-42]	HR [40-42]
Aurantiochytrium sp. SK4[a]	Tubulin [38, 43]	SV40 [38, 43]	HR [38, 43]
Aurantiochytrium sp. YLH70[a]	Actin [56]; ubiquitin [56]	orfC [56]	HR [56]
Parietichytrium sp. TA04Bb	Ubiquitin (*T. aureum*) [27]	Ubiquitin (*T. aureum*) [27]	Random [27]
Schizochytrium sp. S31[a]	35S (CMV) [47, 57, 58]; Tubulin [5, 47]; AlcA (*An*, inducible) [57, 58]; EF1α [48, 53]; EF1α (*Sc*) [48]; ccg1 (Neurospora) [48]; AOX1 (*Pp*, inducible) [48]; Ubiquitin [48]	polyA (CMV) [47, 57, 58]; Nos (*At*) [47, 57, 58]; CYC1 [47]; PFA3 [53]; SV40 [5]; Ubiquitin [48]; AOX1 (*Pp*) [48]	HR [5, 47]; Random [48, 53, 57, 58]
Schizochytrium sp. HX-308[a]	EF1α (*Sc*) [44, 45]; Ubiquitin (n/a) [44, 45]	CYC1 [44, 45]; Ubiquitin (n/a) [44, 45]	HR [44, 45]
Schizochytrium sp. PKU#Mn4[a]	poly-Ubiquitin [46]	CYC1 [46]	HR [46]; Random [46]
Schizochytrium sp. TIO01[a]	EF1α (*Sc*) [49]	CYC1 [49]	HR [49]
Schizochytrium sp. TIO1101[a]	EF1α (*Sc*) [50, 59]; 35S (CMV) [59]	CYC1 [50, 59] ; Nos (*At*) [59]	HR [50]; Random [59]
Schizochytrium sp. SEK 579	Ubiquitin (*T. aureum*) [27]	Ubiquitin (*T. aureum*) [27]	Random [27]
Schizochytrium sp. CB15-5[a]	Actin [51]; EF1α [51]; GAP [51]	Actin [51]; EF1α [51]; GAP [51]	HR [51]

Thraustochytrid strain 12B	EF1α [32]	EF1α [32]	HR [32]
T. aureum ATCC 34304[a]	Ubiquitin [27, 54]; EF-1α [27]; EF-1α (n/a) [54]	Ubiquitin [27, 54]; EF1α [27]; SV40 [54]; EF1α (n/a) [54]	HR [27, 54]; Random [54]
Thraustochytrium sp. ONC-T18[a]	Tubulin [55]	Tubulin [55]	HR [55]

The origins of each element were indicated in parentheses; *Sc*, *Saccharomyces cerevisiae*; All CYC1 originated from *Sc*; All SV40 originated from simian virus 40; Except CYC1 and SV40, all elements without indications are endogenous; EF1α (*Sc*), TEF1; PH, very-long-chain (3R)-3-hydroxyacyl-CoA dehydratase; DH, dehydrase/isomerase; Nos, nopaline synthase; 12B, Thraustochytrid strain 12B; RH-7A, *Aurantiochytrium* sp. RH-7A; SK4, *Aurantiochytrium* sp. SK4; *T. aureum, Thraustochytrium aureum* ATCC 34304; CMV, Cauliflower mosaic virus; *An*, *Aspergillus nidulans*; *Pp*, *Pichia pastoris*; *At*, *Agrobacterium tumefaciens; Hp, Hansenula polymorpha*; n/a, not available; n.r., not relevant (CRISPR-Cas9); [a]With GOIs expressed.

Table S4. The prevalence of constitutive promoters and terminators used in thraustochytrids genetic engineering.

		Non-endogenous			
	**Endogenous**	**Thraustochytrids**	***S. cerevisiae***	**Simian virus 40**	***H. polymorpha***
**Promoter**					
EF1α	4	3	7	0	0
Ubiquitin	4	5	0	0	0
Actin	4	1	0	0	0
Tubulin	4	1	0	0	0
GAP	2	0	0	0	1
**Terminator**					
CYC1	0	0	9	0	0
EF1α	4	2	0	0	0
Ubiquitin	2	4	0	0	0
SV40	0	0	0	5	0
Actin	3	1	0	0	0

The numbers show the number of strains that have used the promoter/terminator in at least one publication (Table S4).

Table S5. Antibiotics used for selecting transformants of thraustochytrids with detailed information regarding to the reference of each concentration.

Strain	Zeocin	Hygromycin	G418	Blasticidin	Other
A. limacinum F26-b		2000 [21-23]	500 [21, 22]		
A. limacinum mh0186	500* [27]	1000 [25]; 2000* [27]	500 [25-28]	1200 [27]	500 (neomycin) [24]
A. limacinum OUC168	5 [29]				100 (chloramphenicol) [29]
A. limacinum OUC88	5 [30]				100 (chloramphenicol) [30]
A. limacinum SR21	30 [35]; 50 [33, 35]; 100 [20, 34, 36, 52]	200 [20]	500 [20, 31, 32]		
Aurantiochytrium sp. KRS101					30 (cycloheximide) [37]
Aurantiochytrium sp. MP4	50 [38]				
Aurantiochytrium sp. PKU#SW7		500 [39]	500 [39]		
Aurantiochytrium sp. RH-7A	100 [36]				
Aurantiochytrium sp. SD116	30 [40]; 50 [42]; 100 [41]	500* [40]	50* [40]; 50 [41]		100* (anhydrotetracycline) [40]
Aurantiochytrium sp. SK4	50 [38, 43]				
Aurantiochytrium sp. YLH70	15 [56]				
Parietichytrium sp. TA04Bb		2000 [27]	500 [27]	800 [27]	
Schizochytrium sp. S31	40 [48]; 50 [5]		100 [47]		50(bleomycin) [47]; 250 (cefotaxime) [57, 58]; 50 (paromomycin) [53]
Schizochytrium sp. HX-308	1.5 [45]; 20 [44]				
Schizochytrium sp. PKU#Mn4			800 [46]		
Schizochytrium sp. TIO01	100 [49]				
Schizochytrium sp. TIO1101			300 [50, 59]		
Schizochytrium sp. SEK 579		2000 [27]	500 [27]		
Schizochytrium sp. CB15-5	20 [51]				
Thraustochytrid strain 12B			500 [32]		
T. aureum ATCC 34304		2000 [27, 54]	1000 [27]; 2000 [54]	200-400 [54]	
Thraustochytrium sp. ONC-T18	250 [55]	400 [55]			

Each number represent the minimal concentration (μg/mL) used on agar for transformant selection in the reference; *Minimum inhibitory concentration identified in the reference that was not used in transformant selection.
